# Supplementary material for: Anonymization of whole slide images in histopathology for research and education
Source: Digit Health. 2023 May 9;9:20552076231171475. doi: 10.1177/20552076231171475 (PMC10185865; doi:10.1177/20552076231171475)
Supplement: sj-docx-1-dhj-10.1177_20552076231171475 - Supplemental material for Anonymization of whole slide images in histopathology for research and education [file sj-docx-1-dhj-10.1177_20552076231171475.docx]

Appendix A

Overview of the metadata categorized as sensitive for each WSI file format.

|  | Leica/Aperio | Hamamatsu | 3DHistech/Mirax | Roche/Ventana | Philips |
| --- | --- | --- | --- | --- | --- |
| Structure | Tiff/BigTiff | Tiff | Configuration file | Tiff/XML | XML |
| Label | x | - | x | x | x |
| Macro | x | x | x | - | x |
| Metadata | ScanScope ID  Date  Time  User  Filename | Macro.S/N  NDP.S/N  Created  Updated | SLIDE_NAME  PROJECT_NAME  SLIDE_ID  SLIDE_CREATIONDATETIME  SCANNER_HARDWARE_ID  SLIDE_UTC_CREATIONDATETIME  ProfileName | JP2FileName  UnitNumber  UserName  Barcode1D  Barcode2D  BaseName  BuildDate | DICOM_ACQUISITION_DATETIME  DICOM_DEVICE_SERIAL_NUMBER  PIIM_DP_SCANNER_OPERATOR_ID  PIM_DP_UFS_BARCODE  PIIM_DP_SCANNER_RACK_NUMBER  PIIM_DP_SCANNER_SLOT_NUMBER |
